# Chronious: the last advances in telehealth monitoring systems

**Published:** 2012-06-15

**Authors:** Giulia Munaro, Roberto Rosso

**Affiliations:** Tesan S.p.A, Italy; Tesan S.p.A, Italy

**Keywords:** chronic disease, patient-professional interfaces, lifestyle

## Abstract

The effectiveness of treatment depends on the patient’s ability to manage in the everyday life his/her chronic health status in accordance with the medical prescriptions outside the hospital settings. For this reason, the European Commission promotes research in tele-health applications, such as Chronious “An Open, Ubiquitous and Adaptive Chronic Disease Management Platform for COPD and Renal Insufficiency”. The aim is the improvement of healthcare service by offering an online health management solution that addresses the patient-professional interaction, personal data security, reduction of hospitalization and related costs. Chronious implements a modular hardware-software system that integrates existing healthcare legacy system, biomedical sensors, user interfaces and multi-parametric data processing with decision support system for patients and health professionals. Nowadays, very few of chronic disease management tools commercially available are accompanied with patient-professional interfaces for communication and education purposes. As added value, Chronious proposes lifestyle and mental support tools for the patients and ontological cross-lingual information retrieval system for clinicians for faster and easier queries to medical knowledge. The patient at home is equipped with a T-shirt able to record cardiac/respiratory/audio and activity signs, external devices (weight scale, glucometer, blood pressure monitoring device, spirometer, air quality sensor) and a touch-screen computer to send reminders on drugs intake and to collect information on dietary habits and mental status. All information are automatically transmitted via IP/GPRS to the Central System, that using a web-interface and ruled based algorithms allows clinicians to monitor patients status and give suggestions for acting in case of worsening trend or risk situation. As consequence, critical procedures that are quite complicated for the patient such as frequent/continuous monitoring, visits to hospitals, self-care are becoming straightforward and simpler. In addition, the information of the clinician is more direct, accurate and complete improving the prognosis for the chronic diseases and the selection of the most appropriate treatment planning. For validation purposes, Chronious is focused on chronic obstructive pulmonary disease and chronic kidney disease, being these widespread and highly expensive in terms of social and economic costs. The validation protocol considers also the most frequent related comorbidities, such as diabetes, involving the patients category which will take advantage of the highest foreseen benefits. This enables an open architecture for further applications. Project validation is divided in two progressive phases: the first one in hospital setting was aimed to verify on 50 patients if the delivered prototypes met the user requirements, the ergonomic and functional specifications. The second phase has observational features. The improved system is currently applied at home on 60 selected patients. Patients are instructed to use the system independently for an expected duration of 4 months each. In parallel, the patient is monitored with standard periodic outpatient checks. At the end, customer satisfaction and the predictive ability of the system in the evolution of the disease will be evaluated. First feedbacks are encouraging because Chronious monitoring provides friendly approaches to new technologies and reassures patients reducing the intervention time in critical situation.

